# Conductivity experiments for electrolyte formulations and their automated analysis

**DOI:** 10.1038/s41597-023-01936-3

**Published:** 2023-01-19

**Authors:** Fuzhan Rahmanian, Monika Vogler, Christian Wölke, Peng Yan, Stefan Fuchs, Martin Winter, Isidora Cekic-Laskovic, Helge Sören Stein

**Affiliations:** 1 Helmholtz Institute Ulm, Applied Electrochemistry, Helmholtzstr. 11, 89081 Ulm, Germany; 2 Karlsruhe Institute of Technology, Institute of Physical Chemistry, Fritz-Haber-Weg 2, 76131 Karlsruhe, Germany; 3 Helmholtz Institute Münster (IEK-12), Forschungszentrum Jülich GmbH, Corrensstraße 46, 48149 Münster, Germany; 4 MEET Battery Research Center, University of Münster, Corrensstrasse 46, 48149 Münster, Germany

**Keywords:** Batteries, Characterization and analytical techniques

## Abstract

Electrolytes are considered crucial for the performance of batteries, and therefore indispensable for future energy storage research. This paper presents data that describes the effect of the electrolyte composition on the ionic conductivity. In particular, the data focuses on electrolytes composed of ethylene carbonate (EC), propylene carbonate (PC), ethyl methyl carbonate (EMC), and lithium hexafluorophosphate (LiPF_6_). The mass ratio of EC to PC was varied, while keeping the mass ratio of (EC + PC) and EMC at fixed values of 3:7 and 1:1. The conducting salt concentration was also varied during the study. Conductivity data was obtained from electrochemical impedance spectroscopy (EIS) measurements at various temperatures. Based on the thus obtained temperature series, the activation energy for ionic conduction was determined during the analysis. The data is presented here in a machine-readable format and includes a Python package for analyzing temperature series of electrolyte conductivity according to the Arrhenius equation and EIS data. The data may be useful e.g. for the training of machine learning models or for reference prior to experiments.

## Background & Summary

Electrolytes are crucial for the performance of batteries^1^ since they enable shuttling of the ions, provide electrical isolation of the electrodes and have a defining influence on the formation and stability of the solid electrolyte interface (SEI)^2^ and the cathode electrolyte interface (CEI)^2–4^. Achieving high performance electrolytes, typically requires the presence of various components like organic solvents, co-solvents, functional additives and conducting salts^5^. The concentration of each component and the ratio between the components have a strong impact on the conductivity of the electrolyte^6–8^. Ding *et al*. showed in several studies^6–9^, that the composition of the electrolyte, especially the PC content, affects the viscosity and glass transition temperature of the electrolyte. The amount of PC also hinders crystallization of EC^6,10^. This allows for the formulation of electrolytes with improved performance at low temperatures^10,11^.

The dataset^12^ presented herein provides a comprehensive basis for future optimization studies, as it contains a wide variation of formulations and temperatures, including the raw data. Furthermore, it can help to gain deeper insights regarding composition-property-performance relationships. Fractions of this dataset served as the basis for several machine learning models published elsewhere^11,13,14^. The automated high-throughput experimentation system^13^ available at the Helmholtz Institute Münster is used to formulate a variety of electrolyte solutions based on EC, EMC, PC and LiPF_6_. Ratios of (PC + EC):EMC of 3:7 and 1:1 are covered in the dataset^12^. The concentration of the conducting salt varies between 0.2 mol kg^−1^ and 2.1 mol kg^−1^, while the ratio of EC:PC ranges from 0.0 to 9.2.

**Fig. 1:**
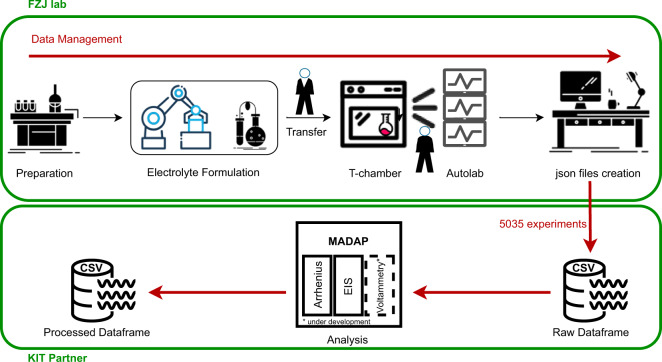
The overall workflow representation from experimentation to data generation in Helmholtz-Institute Münster and data analysis in Karlsruhe Institute of Technology (KIT) and its partners.

The robotic system^13^ used for the acquisition of the data is able to dispense liquid and solid components into aluminium or polymer vials with high accuracy. Each formulation is identified by a batch number and measurements are identified by a unique ID stored and reported on the vial through a QR code. After sample-preparation, the automated setup performs the targeted measurement. Subsequently, the system returns a JSON formatted file for each formulation, which allows for downstream processing. Here, we present the data^12^ as a CSV file to summarize the results received from 504 individual JSON files. Manual analysis of the raw data is time intensive, which is why we have developed an automated Python-based data analysis package called Modular and Autonomous Data Analysis Platform (*MADAP*)^15^ with a command line interface (CLI) and a graphical user interface (GUI) that can process the aggregated CSV. This package is generalized and can be used on a variety of datasets as described below. The overall workflow of generating and analyzing data is shown in Fig. 1. All input parameters are tracked and saved in the output obtained from *MADAP*^15^ to allow full data provenance tracking^16,17^ of not just the experimental but also the data analysis steps in the research workflow^18^.

The dataset^12^ can be used to train machine learning models in order to predict promising electrolyte formulations to reach an optimum conductivity, as demonstrated by Rahmanian *et al*.^11^. Further, the research community may find the data useful in the design of their own experiments and in decisions concerning the use of hardware, software and human resources. The use of this dataset together with analysis tools like *MADAP*^15^ as a base for further lithium-ion battery research, enables the generation of further insights such as the activation energy of the ion conduction process. It is even possible to add other analysis procedures to *MADAP*^15^ to further expand the automation it provides.

## Methods

### High throughput experimentation (HTE) system

The robotic HTE system^13^, used to acquire the data^12^ presented here, is designed for high-throughput operation in a nitrogen atmosphere. The setup designed for the formulation of electrolyte solutions is able to prepare 96 formulations in 8 h by gravimetric dosing of solid and liquid materials into polymer or aluminium vials. Up to 10 mL of electrolyte can be formulated within one vial. The setup also provides functionalities to close the vials, mix, and heat their content using a heated shaker plate. Further, EIS measurements are performed automatically. To track the samples, each vial is automatically labelled using a QR code representing information like the date of preparation, an ID for the electrolyte mixture and information regarding the chemicals used. In preparation for EIS measurements, a volume of 750 μL of the electrolytes is automatically filled into single-use Eppendorf ^Ⓡ^ Safe-Lock Tubes with a capacity of 2 mL. The use of single-use equipment avoids cross contamination in this step of the process. Subsequently, electrodes are automatically immersed into the sample. These electrodes are designed to generate reproducible results independent of the shape of the vial or the depth of immersion^19^. For the measurement, the samples are arranged in groups of eight samples per rack, three of which are mounted on one larger rack. Four of these combined racks can be connected to the *Metrohm Autolab* potentiostat, which is used for the measurements^13^.

### EIS measurement

After the assembly of the racks, they are manually transferred to a *Memmert TTC256* temperature chamber for EIS measurements. The connection of the cells to the *Metrohm Autolab* potentiostat is also done by the operator. The temperature chamber is programmed such, to cover the temperature ranges between −30 °C and 60 °C in steps of 10 °C. Subsequent to an equilibration period of 2 h for each temperature, the EIS measurements are automatically performed with an applied AC voltage of 40 mV and frequencies between 20 kHz to 50 Hz. A multiplexer distributes the output of twelve channels to eight outputs each. Hence, 96 channels are available to connect to each of the 96 cells on a rack^13^. Each experiment is repeated several times to provide up to 8 sets of values to the dataset. Repetitions can be identified and distinguished based on the running number in the experimentID.

### Data management in the experimental setup

The data recording during the experimental workflow is handled by a laboratory information management system. It records identifiers for the starting materials, test protocols and relevant experimental parameters. Furthermore, the system is able to merge these data with metadata comprising further details about the electrolytes used in a measurement. After conclusion of a measurement, the collected data including the metadata is saved to a JSON file, which can be used for analysis.

### Analysis Software (*MADAP*)

**Fig. 2:**
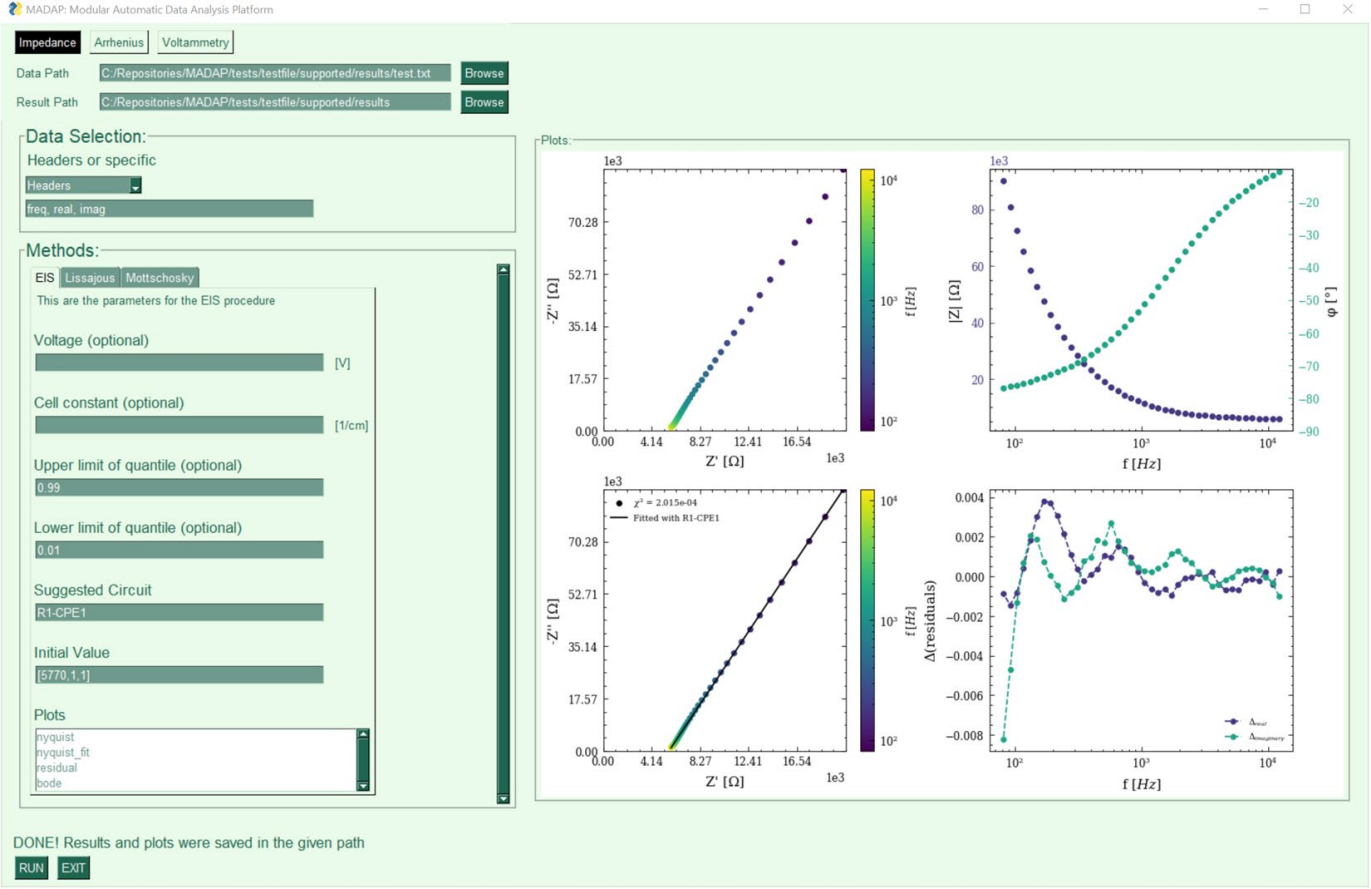
A showcase of the Graphical User Interface diagram of *MADAP*^15^.

For the data analysis, a variety of tools are present and available, e.g. ZView^20^, pyEIS^21^, impedance^22^, Aftermath^23^ and Origin^24^. We decided to bundle some of these tools into a compact, modular software package called *MADAP*^15^, thoroughly documented using sphinx^25^. This analysis tool provides all the necessary means to perform electrochemical data analysis based on experimental datasets, while providing full data provenance tracking, and plot publication quality results. It can perform a variety of automated electrochemical analyses, including EIS, linear and cyclic voltammetry and the analysis of temperature series according to the Arrhenius equation. In this paper, we focus on Arrhenius analysis and EIS measurements. *MADAP*^15^ is deployed in Python3 and is publicly accessible as a GitHub repository (https://github.com/fuzhanrahmanian/MADAP)^15^, a pip installable package (pip install madap), and an executable (https://github.com/fuzhanrahmanian/MADAP/releases/tag/v1.0.0) with a graphical user interface (GUI) created with PySimpleGui^26,27^, as shown in Fig. 2. The accessibility of *MADAP*^15^, by means of a CLI as well as a GUI, provides the broader scientific community with a variety of entry points for the data analysis. The generic nature of the procedure assures that the package can be expanded with further analysis methods without impacting the existing methodologies. Further, this enables its integration into autonomous research workflows^28–30^. The basic workflow of an analysis using *MADAP*^15^ comprises the three steps of data acquisition, pre-processing and the analysis itself. In the former, the user can import different data types (.txt, .json, .hdf5 or .h5, .xml, .pkl and .csv) and select the data to be analyzed based on ranges of indices for rows and columns or by specifying column labels. The pre-processing step can detect outliers based on given upper and lower limits of the relevant quantile using the *Quantile-based flooring and capping* algorithm^31^. The user may choose to specify custom limits or use the default values implemented in *MADAP*^15^. In version 1.0, the default values are chosen as 0.01 for the lower and 0.99 for the upper limit. Afterwards, the user can choose what type of analysis shall be performed, i.e. voltammetry, EIS or Arrhenius.

**Fig. 3:**
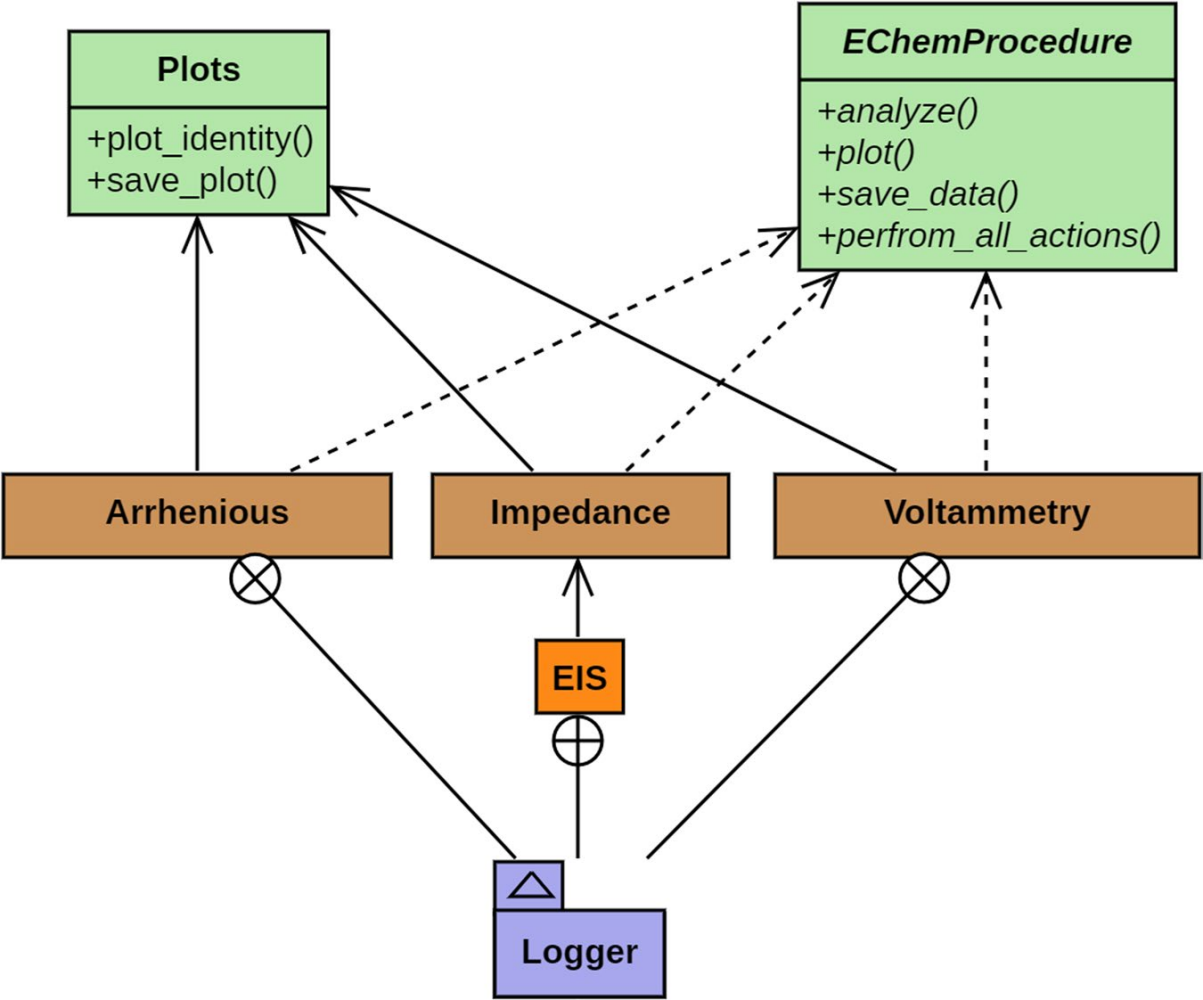
The stylized Unified Modelling Language (UML) diagram that represents the code structure of *MADAP*^15^.

Figure 3 depicts the code structure used in *MADAP*^15^. In the beginning of each analysis, all the procedures instantiate an abstract class called EChemProcedure, which enforces the presence of methods called analyze, plot, save_data and perform_all_actions. All procedures additionally inherit from the common Plots class, which equips them with the common plotting functionalities, providing outputs with scientific format^32^. The complete procedure is continuously logged to review potential errors.

The linear fit required for the Arrhenius type analysis^33^ is implemented in *MADAP*^15^ using the functionalities for linear regression provided in the *scikit-learn* package^34^. The activation energy and the pre-exponential factor are derived from this fit. The regression loss, which is chosen as a quality metric, is calculated using the mean square error (MSE). Finally, plots and data files for the raw and fitted data as well as the model’s parameters are automatically generated and saved in a designated location in accordance to the FAIR (Findability, Accessibility, Interoperability, and Reusability) data principle^35^.

EIS analysis and fitting are performed by a partial adoption of the *impedance* package provided by Matthew D. *et al*.^22^. In this package, the model uses a non-linear square fit as supplied by the *SciPy*^36^ package. The EImpedance module of *MADAP*^15^ gives the user the possibility to provide a definition of an equivalent circuit *via* available elements and their corresponding values. In this case, the user should provide guesses for the value of each element in the selected circuit. Based on these guesses, *MADAP*^15^ generates a fit of the selected data internally and evaluates its quality. For the quality check, the root-mean-square error (RMSE) of the fit is determined and compared to the root mean square (RMS) of the experimental data. If the ratio of RMSE over RMS exceeds a threshold (*δ*), a re-evaluation will be triggered. In this case, the standard deviation of each estimated value of a circuit’s element is added to or subtracted from the respective value to improve generalization. The operation to be carried out is selected randomly for each value. These new values are then used as the input guesses for the subsequent fit. This procedure is iterated, until either the ratio of RMSE and RMS is below *δ*, i.e. Equation 1 is fulfilled, or 5 iterations are reached. The number of iterations as well as *δ* are determined heuristically to 5 and 0.01, although the user will have the possibility to change them and define custom numbers as required.

1 $$0\le \frac{RMSE}{RMS} < \delta $$

**Fig. 4:**
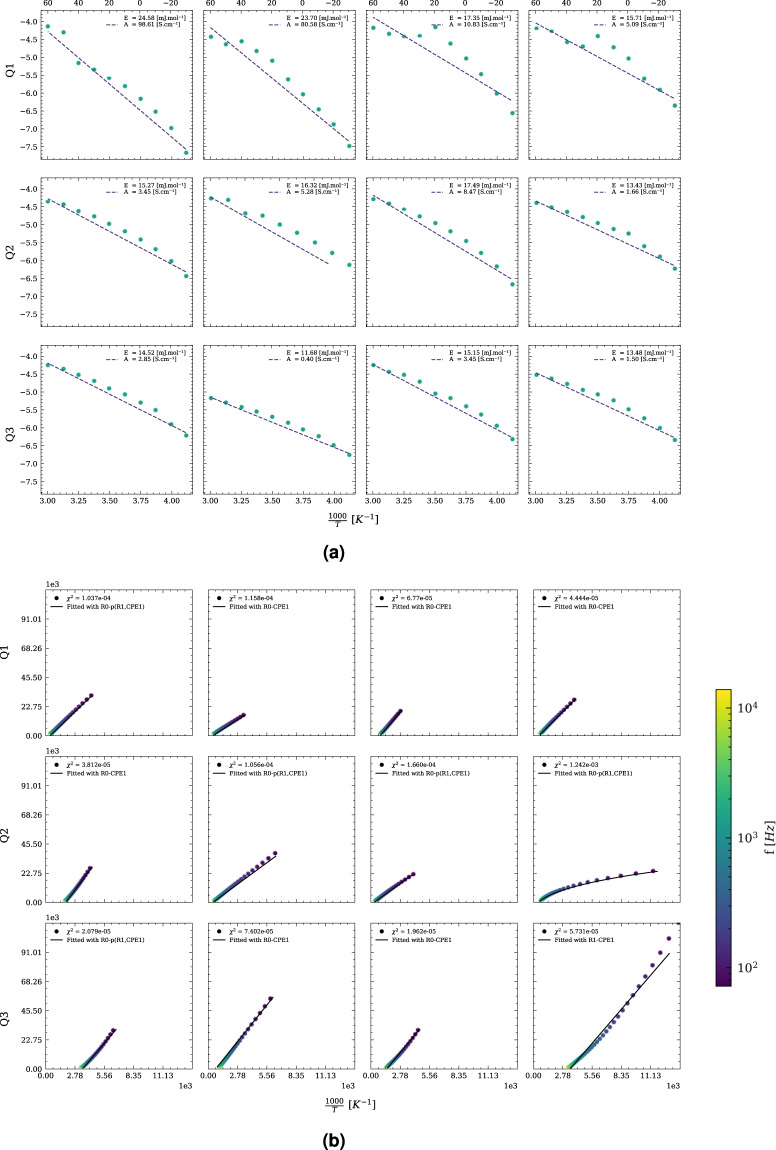
Fits randomly selected from Q1, Q2, Q3 based on the (**a**) *R*^2^ score of the Arrhenius fit and (**b**) RMSE of the eis fit determined by *MADAP*^15^ for 5035 electrolyte measurements with a frequency range between 50 and 20k.

Alternatively, *MADAP*^15^ provides the option to iterate over 40 common hard-coded equivalent circuits, which are provided as part of the *MADAP*^15^ package, without further user input. In this case, the match with the lowest RMSE will be chosen. This metric will be used as the loss metric in the analysis. For every impedance spectrum, the fitted circuit parameters and their uncertainties, the loss metric, the determined resistance and the corresponding conductivity will be saved automatically. To provide information about the linearity and stability of the fit, the improved *linear Kramers-Kronig* (linKK) method^37^ as implemented in the *impedance* module^22^ is applied automatically to each spectrum. For visualization, a Nyquist and a Bode plot comprising the raw and fitted data as well as a residual plot for the linKK method will be generated and saved accordingly. Figure 4b shows the data and the fit of randomly selected spectra corresponding to different quantiles of the RMSE to convey an impression of the achieved quality of the fit. For each quantile, four spectra and their respective fits are shown. For evaluation of the reliability of the fit, benchmarking is done referencing to the manual analysis of the selected data using *Metrohm Autolab* software as a baseline. In comparison to this baseline, *MADAP*^15^ provides acceptable fits for the majority of the spectra. The same principle was applied for the Arrhenius analyses, depicted in Fig. 4a.

## Data Records

The dataset^12^ presented here comprises, among others, conductivity, real and imaginary part of the impedance as determined by EIS measurements and information regarding the formulation of a variety of electrolyte formulations for lithium-based batteries. The formulations relate to the masses of the solvent components EC, PC, and EMC and the conducting salt LiPF_6_.

We provide the dataset as a dataframe in a CSV file format, which can be dowloaded from 10.5281/zenodo.7244939^12^ and may be used under the CC BY license. A summary of its structure is presented in Table 1. This table also shows the data type, the range of values covered for each quantity, the number of unique values and the physical unit. In this section, we elucidate more on the data and the interrelations within the dataframe.

**Table 1:** This table describes the data comprised in the dataset presented herein.

Column name	Description	Data type	Range	Unique entries	Unit
experimentID	A unique identifier for each experiment coding an operator, the date of the experiment and the batch of the electrolyte used.	string	not applicable	504	—
temperature	The temperature at which the measurement was conducted	float	(−30, 60)	10	°C
frequency	A series of frequency values selected for the electrochemical impedance spectroscopy	Str[List[float]]	(50, 20000)	1	Hz
real_impedance	A series of the real part of the impedance measured by means of the electrochemical impedance spectroscopy	Str[List[float]]	(−390106, 114305526)	5035	Ω
imaginary_impedance	A series of the imaginary part of the impedance measured by means of the electrochemical impedance spectroscopy	Str[List[float]]	(−371850382, 103)	5035	Ω
cell_constant,_standard_deviation	A tuple comprising the cell constant and its standard deviation determined from five measurements using a 0.01 M KCl (aq) standard solution at 20 °C with a 2 h equilibration period between measurements	Str[Tuple[float]]	(3.815, 4.720); (0.000, 0.178)	339	cm^−1^
PC	The mass of propylene carbonate (PC) used for electrolyte formulation	float	(0.273, 5.306)	105	g
EC	The mass of ethylene carbonate (EC) used for electrolyte formulation	float	(0.000, 4.320)	99	g
EMC	The mass of ethyl methyl carbonate (EMC) used for electrolyte formulation	float	(5.293, 9.457)	105	g
LiPF_6	The mass of lithium hexafluorophosphate (LiPF_6_) used for electrolyte formulation	float	(0.301, 4.093)	100	g
metadata	Further metadata regarding the electrolyte solution arranged in a dictionary with the keys experimentDate, experimentType, formatVersion, channel, electrolyteAmount, suspectedMeasurementError, PC, EC, EMC, and LiPF_6_	Str[Dict[str]]	not applicable	504	—
phase_shift	The phase shift as obtained from EIS analysis as implemented in *MADAP*	Str[List[float]]	(0.131, 89.882)	5035	°
EIS_conductivity	The conductivity as obtained from EIS analysis performed using *MADAP*	float	(0.000, 0.019)	5035	S cm^−1^
EIS_fittedParameters	The values and corresponding uncertainties of the elements in the equivalent circuit as determined using *MADAP*	Str[List[tuple]]	not applicable	5035	—
EIS_RMSE	The RMSE of the fit obtained by applying the equivalent circuit determined using *MADAP* in the real and imaginary dimension	float	(4.363, 28560.795)	5035	—
EIS_numberRCelements	The required number of RC elements in the equivalent circuit required to reproduce the EIS spectrum determined using the linKK method as implemented in the *impedance* package^22^	float	(5, 11)	7	—
EIS_fitEvaluation	A numeric value indicating the quality of the fit. A value close to unity indicates a good fit.	float	(0.576, 0.850)	5035	—
EIS_resistance	The ionic charge transfer resistance as obtained from EIS analysis as implemented in *MADAP*	float	(241.781, 25564.121)	5035	Ω
EIS_chiSquare	A statistical measure for the goodness of the fit as obtained from the linKK method as implemented in the *impedance* package^22^	float	(0.000, 0.322)	5035	—
EIS_circuit	The equivalent circuit for the EIS spectrum as obtained from the linKK method as implemented in the *impedance* package^22^	string	not applicable	8	—
EIS_impedance	A list of impedance values obtained from the fit generated during the EIS analysis performed using *MADAP*	Str[List[compex]]	not applicable	5035	Ω
EIS_residualReal	The real part of the residuals of the fit as determined using the linKK method as implemented in the *impedance* package^22^	Str[List[float]]	(−0.118, 0.170)	5035	Ω
EIS_residualImaginary	The imaginary part of the residuals of the fit as determined using the linKK method as implemented in the *impedance* package^22^	Str[List[float]]	(−0.118, 0.170)	5035	Ω
Arrhenius_activationEnergy	The activation energy obtained from the analysis according to the Arrhenius equation using *MADAP*	float	(9.427, 30.413)	504	mJ mol^−1^
Arrhenius_preExponential	The pre-exponential factor obtained from the analysis according to the Arrhenius equation using *MADAP*	float	(0.109, 962.145)	504	—
Arrhenius_R2	The *R*^2^ score corresponding to the linear fit obtained in the analysis according to the Arrhenius equation using *MADAP*	float	(0.186, 0.999)	504	—
Arrhenius_MSE	The mean square error for the linear fit determined during the analysis according to the Arrhenius equation using *MADAP*	float	(0.000, 0.703)	504	—
Arrhenius_lnConductivity	A list of *Inσ* obtained from the linear fit according to the Arrhenius equation determined by *MADAP*	float	(−8.175, −3.713)	5035	ln(S cm^−1^)

The robotic system operated at the Helmholtz Institute Münster outputs the raw data in JSON format. Although, this format is machine-readable, we decided to provide the data in CSV format, which can easily be read into the user’s script as a table, e.g. using the Pandas^38^ library available for Python. Each line in the dataframe represents all the data available for a single measurement. Parameters, which are shared by several experiments, are repeated in each line, where they are applicable. In the following, we will elucidate more on each column of the dataframe.

### experimentID

This column provides a unique identifier for each experiment, which enables traceability of the data. It codes the operator, the date of the experiment, the label of the electrolyte and a running number differentiating the repeats. The format of the experimentID is: [operator]_[date of the experiment]_[label of the electrolyte]_[running number].

### temperature

The temperature, at which each measurement was performed, is reported in this column. Each row corresponds to a measurement at one temperature. The values range from −30 °C to 60 °C. For five formulations, the measurement at −30 °C is not reported in the dataset.

### frequency

This column reports a string, which comprises a list of the frequencies used in the EIS measurements. The frequencies are reported in units of Hz and cover a range from 20 kHz to 50 Hz.

### real_impedance

Values for the real part of the impedance, Z’, in the unit Ω are given in this column in the form of a string of a list of floats. The values in this column for all measurements range from −3.901 × 10^5^ Ω to 11.430 × 10^7^ Ω. The negative values result from artefacts in the measurements.

### imaginary_impedance

The imaginary part of the impedance, Z”, is presented in this column. The values are given in Ω and range from −37.185 × 10^7^ Ω to 103.002 Ω. The positive values result from artefacts in the measurements.

### cell_constant,_standard_deviation

The cell constant and the respective standard deviation values are reported in cm^−1^ and determined from five reference measurements using 0.01 M KCl (aq) standard solution at a temperature of 20 °C^13^. In the dataset, they are reported in a common column as a tuple, in which the first value corresponds to the cell constant and the second value reports the standard deviation. The values for the cell constant range from 3.815 to 4.720, while the standard deviations span a range from 0.000 to 0.178.

### PC

This column reports the mass of PC in g used during the preparation of the electrolyte formulation. The values are given as floats and range from 0.273 g to 5.306 g.

### EC

The mass of EC used during the preparation of the electrolyte formulation is reported in this column. The values are given as floats in units of g and are spanning a range from 0.000 g to 4.320 g.

### EMC

In this column, we report the mass of EMC used for the preparation of the electrolyte formulation. The values are given in g and comprise values between 0.480 g and 9.457 g.

### LiPF_6

This column presents the mass in g of LiPF_6_ comprised in the formulations. The values reach from 0.301 g to 4.093 g.

### metadata

In this column, additional information is reported, which cannot be reasonably presented in tabular form. The metadata are presented as a string of a dictionary. It reports the date and type of the experiment using the keys *experimentDate* and *experimentType*, respectively. Further, the version of the JSON format is associated with the key *formatVersion*. The number of the channel running the experiment, the amount of electrolyte used in the respective measurement, and the suspected measurement error are correlated with the keys *channel*, *electrolyteAmount*, and *suspectedMeasurementError*, respectively. The keys *PC*, *EC*, *EMC*, and *LiPF*_6_ are linked to further information regarding the respective electrolyte component which is represented in dictionary format. The keys *Batch-No*, *CAS-No*, and *comment* present the respective information as a string. The date of delivery and the date of opening of the container are given as strings in the format MM/YY and can be accessed using the keys *dateOfDelivery* and *dateOfOpening*. The molar mass of the substance is reported as a float with the key *molarMass*, while its unit is given as a string using the key *molarMassUnit*. The *name* key is associated with a string stating the long name of the chemical. The purity of the material is found using the key *purity*, while the SMILES string is given with the key *SMILES*. Both of these quantities are reported as strings. The amount of the respective substance used in the formulation is accessed with the key *substanceAmount*, while the respective unit is found using the key *substanceAmountUnit*. Finally, the *supplier* key returns the supplier, from which the material was obtained.

Moreover, the dataframe also contains data resulting from the analysis of the experimental data using the *MADAP*^15^ Python package. The *MADAP*^15^ analysis workflow is performed on a Lenovo Workstation with an AMD Ryzen Threadripper PRO 3975WX processor at 3500 MHz with 32 cores and 64 Logical Processors. The workstation is equipped with 128 GB of RAM and an RTX A6000 GPU running with Microsoft Windows 10 Pro. The single core performance of the CPU turned out to be a bottleneck during operation, since the used libraries are not optimized for multicore processing or GPU training. Hence, *MADAP*^15^ was configured to use all 32 cores for multithreaded operation for this scenario. In the following, we elucidate more on the analyzed results contained in the dataframe by going through the column names associated with analyzed data.

### phase_shift

This column reports the phase shift (*ϕ*) or phase angle as obtained from the EIS analysis implemented in the *MADAP*^15^ package according to Eq. 2:

2 $$\phi =\arctan \left|\frac{Z{\prime\prime} }{Z{\prime} }\right|.$$

The data is given as a string of a list with values ranging from 0.131 to 89.882 given in°.

### EIS_conductivity

The ionic conductivity obtained as the quotient of the cell constant and the resistance determined from the EIS analysis implemented in *MADAP*^15^ is reported in this column. The conductivity is given in units of S cm^−1^ and the values range from 0.000 S cm^−1^ to 0.019 S cm^−1^.

### EIS_fittedParameters

In this column, we report the determined values of the circuit’s elements as well as their uncertainties as obtained from the analysis. These parameters are represented as a string of a list of tuples. The first element of each tuple illustrates the value of the respective element, and the second value shows the standard deviation error obtained from the output of the *impedance* package^22^. The order of the tuples corresponds to the order of a given circuit’s elements as presented in column EIS_circuit.

### EIS_RMSE

This column reports the RMSE of the fit in the real and the imaginary dimension as obtained from EIS analysis. The values are given as floats.

### EIS_numberRCelements

An optimal number of RC elements in an equivalent circuit determined using the linKK method can be verified by a metric, which subtracts the ratio between the sums of negative and positive resistor values from unity. The symbolic representation of this metric is conventionally chosen to be *μ* and its values are reported as floats in our dataframe. The number of RC elements considered as optimal is the one, which results in a value of *μ* below 0.85^37^.

### EIS_fitEvaluation

This column reports a numeric value providing means to estimate the degree of over- or under-fitting. The values range from 0.576 to 0.850 and are reported as floats. The upper limit is fixed at 0.850 to avoid overfitting, as described by Schönleber *et al*.^37^.

### EIS_resistance

From the EIS analysis, the resistance of the electrolyte towards ionic charge transfer is obtained. The values resulting from the analysis are reported in this column in units of Ω. A range from 241.781 Ω to 25.564 × 10^3^ Ω is spanned by the data.

### EIS_chiSquare

This statistical value determines the goodness of the fit derived from the linKK method and is calculated as the sum of squares of the real and imaginary residual error. The *χ*^2^ values are reported as floats.

### EIS_circuit

The manual or auto-selected circuit used to fit the EIS data of the concerned measurement is reported in this column. In the representation, serial connections are displayed as *element*_1_*-element*_2_, while *p(element*_1_*, element*_2_) indicates a parallel electric connection. The elements in the circuit are represented by *R* for resistance and *C* for capacity. A constant phase element is indicated by *CPE* and a Warburg element is represented as *W*. An additional list of elements, which may be used by the user, can be found in the *impedance* package^22^. In this column, the fitted circuit for each conductivity experiment is represented by a string.

### EIS_impedance

This column represents a list of impedance values obtained from the fitted model with frequency as input and the measured impedance as output. The data is reported as a string of a list.

### EIS_residualReal

The residual errors of the real impedance obtained from the linKK method can be seen in this column. They are given as a string of a list.

### EIS_residualImaginary

In this column, the residual error derived from the linKK method for imaginary impedance as a consistency factor is reported as a string of a list.

### Arrhenius_activationEnergy

For calculating the activation energy from the conductivity experiment, a linear fit between the inverse temperatures in 1000/K and the natural logarithm of conductivities is applied. The activation energy can be calculated with the Arrhenius equation and is reported as a float in this column with the unit mJ mol^−1^.

### Arrhenius_preExponential

The pre-exponential factor obtained from the linear fit according to the Arrhenius equation is reported in this column. The values of this factor are given as a float with the unit Scm^−1^.

### Arrhenius_R2

In this column, the R^2^ score of the linear fit is shown as a unitless float.

### Arrhenius_MSE

In this column, we report the mean square error of the linear fit as a unitless float.

### Arrhenius_lnConductivity

A list of the natural logarithmic conductivities obtained from the linear fit is reported in this column as a string of a list of floating point numbers.

**Fig. 5:**
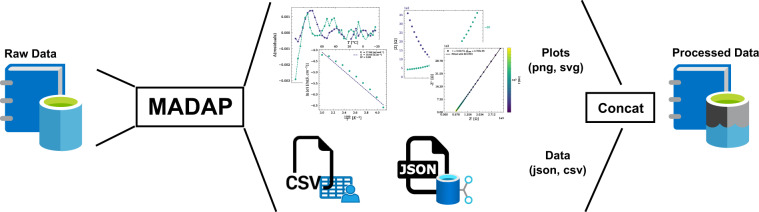
Schematic representation of the given dataframe consisting of raw and processed data.

All the relevant data concerning the raw data, fitting parameters and results of the analysis are saved in the presented dataset. The data is therefore fully traceable and reusable. This is compliant with the FAIR^35^ data standard. The workflow is schematized in Fig. 5.

The column named *Data Type* in Table 1 shows the data type obtained after reading the dataframe from the CSV file using Pandas’^38^. read_csv method. The user should note the information provided in the column *Description* to see the structure of the string. For example, the real part of the impedance is read as a string type variable. However, it actually represents a list of floats and should be cast to this data type.

## Technical Validation

The reliability of the experimental data is validated by repeating each measurement several times. Invalid data is not stored in the dataset^12^ reported here. Each measurement is examined by an expert in the field to ensure high quality of the data.

The data obtained from the analysis is verified using an appropriate metric for each analysis. For the Arrhenius type analysis, the quality of the fit is quantified by the mean squared error (MSE).

The impedance data reported in this work is pre-processed for analysis by excluding negative impedance values and outliers to enable a reliable analysis. The linKK method is used to verify the linearity of the spectrum and also reports the goodness of the fit by the statistical *χ*^2^ value corresponding to the residual errors of the impedance data. Consequently, the resulting fit of the equivalent circuit is verified by means of the RMSE. This workflow returns the parameters corresponding to the equivalent circuit as presented in the section Data Records.

**Fig. 6:**
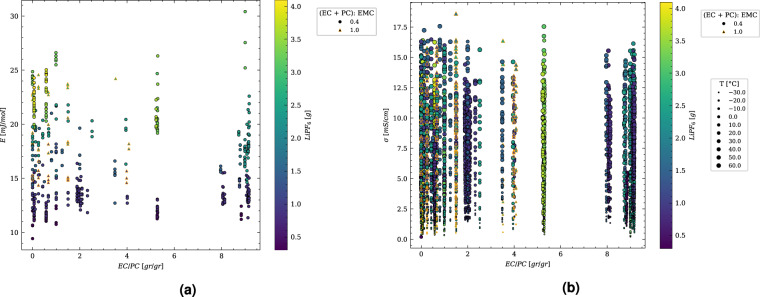
Results from the analysis according to the Arrhenius equation. (**a**) represents the activation energies for two (EC + PC):EMC ratios of 0.4 and 1, derived by *MADAP*^15^ using a linear regression fit, (**b**) shows the conductivity value for the mentioned ratios at 10 descrete temperatures between −30.0 °C and 60.0 °C obtained from the analysis performed by *MADAP*^15^ using non-linear least square fit of SciPy^36^ module. For a part of this fit, the impedance module^22^ has been utilized.

For visualization, we generated quantiles based on R^2^ and RMSE for all the fits performed during the analyses. Figure 4 shows the results of four randomly selected analyses taken from each quantile to provide an overview of the distribution of the fitting quality. In Fig. 4a, fits corresponding to quantiles based on R^2^ are shown, while Fig. 4b presents fits for quantiles based on RMSE. The first row in each subfigure gives an impression of the lowest fit quality, while the best fits are shown in the last row of the subfigures. Additionally, the conductivity and the activation energy calculated by *MADAP*^15^ are depicted in Fig. 6.

## Usage Notes

It is recommended to apply the *MADAP*^15^ package to use, extend or adapt the provided data analysis. For performing analysis using the *MADAP*^15^ package, a specific range of rows and columns of the dataframe can be selected. For example, to reproduce one of the result of this article for Arrhenius analysis, the published dataset was selected as input for the *MADAP*^15^ GUI and the row indices from 3967 to 3977 and column 2 for temperatures and column 13 for electrolyte conductivity selected for the evaluation. Both plotting types were chosen, and the RUN button was pressed. Further results can be derived similarly.

Note that, in case a definition of the formulation in terms of molar fractions is desired, the amounts of substances for each component of the electrolyte as reported in the dictionary given in the column labelled metadata can be used.

## Data Availability

The code of the *MADAP*^15^ package is publicly available on https://github.com/fuzhanrahmanian/MADAP and the documentation can be found in https://fuzhanrahmanian.github.io/MADAP/. A stand-alone windows executable can be downloaded from the GitHub repository as well. Furthermore, *MADAP*^15^ can be installed by running pip install madap. The analysis results presented in this article are generated using *MADAP*^15^ version 1.0. Contributions are welcome, but should follow the common guidelines for group software development, which can be found in the CONTRIBUTION section of the *MADAP*^15^ the repository. The code is developed for the Python version 3.9 and above and should use the following packages and versions: attrs > = 21.4.0, matplotlib > = 3.5.3^39^, numpy > = 1.22.4^40^, pandas > = 1.4.2^38^, pytest > = 7.1.2, scikit_learn > = 1.1.2, and impedance > = 1.4.1^22^. For running the GUI, PySimpleGUI > = 4.60.3^26^ is required additionally.
